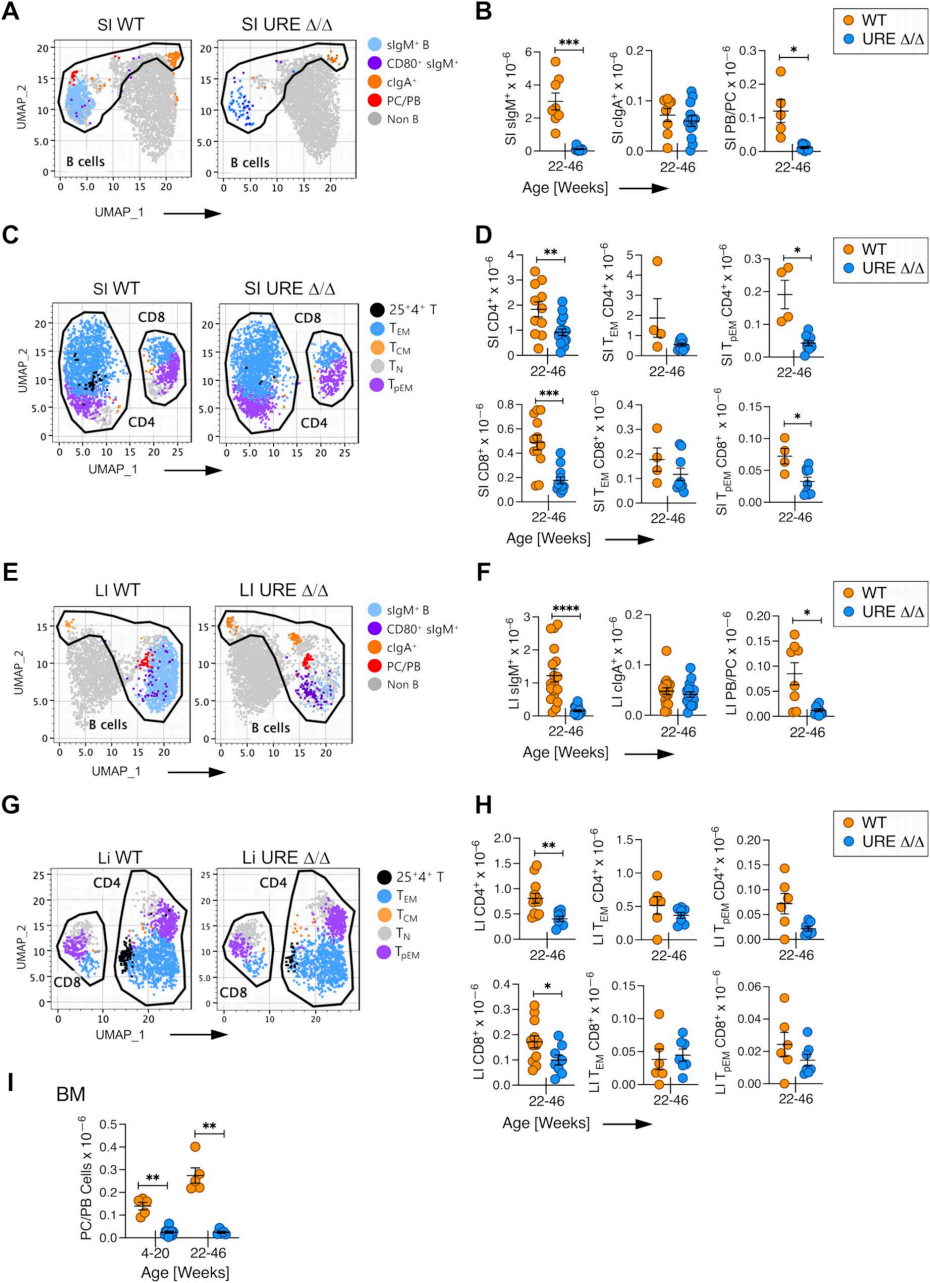# Correction: Transient PU.1 low fetal progenitors generate lymphoid progeny that contribute to adult immunity

**DOI:** 10.26508/lsa.202503567

**Published:** 2025-12-02

**Authors:** Encarnacion Montecino-Rodriguez, Oscar I Estrada, Kenneth Dorshkind

**Affiliations:** Department of Pathology and Laboratory Medicine, David Geffen School of Medicine at UCLA , Los Angeles, CA, USA

## Abstract

Transient PU.1^low^ fetal progenitors generate activated and memory T and B cells that colonize and are maintained in secondary lymphoid tissues and make an early and long-term contribution to the adult immune system.

Article: Montecino-Rodriguez E, Estrada OI, Dorshkind K. Transient PU.1 low fetal progenitors generate lymphoid progeny that contribute to adult immunity. Life Sci Alliance 7(8): e202402629. doi: https://doi.org/10.26508/lsa.202402629. PMID: 38830768.

Following publication, the authors noted that place-holder panels in Fig 6G were not replaced with the correct UMAPs at the time of review and when the final manuscript was submitted for publication. The correct version of this figure has now replaced the older version. The UMAP data shown in Fig 6G are just a visual representation of the quantification data presented in panel 6H, and the formatting error did not change any of the conclusions.

**Figure:**