# Supplementary material for: Transient PU.1 low fetal progenitors generate lymphoid progeny that contribute to adult immunity
Source: Life Sci Alliance. 2024 Jun 3;7(8):e202402629. doi: 10.26508/lsa.202402629 (PMC11147949; doi:10.26508/lsa.202402629)
Supplement: Supplementary file 1 [file LSA-2024-02629_TableS1.docx]

**Table S1** Antibodies, clone numbers used in this study and their source.

| **Antibodies for immunostaining** | **SOURCE** | **Cat #** |
| --- | --- | --- |
| Fixable Viability Dye eFluor™ 780 | ThermoFisher Scientific | 65-0865-14 |
| Purified CD16/32 (Clone: 93) | ThermoFisher Scientific | 14-0161-86 |
| CD21/CD35- FITC (Clone: 7G6) | BD Biosciences | 553818 |
| CD62L- FITC (clone: MEL-14) | ThermoFisher Scientific | 11-0621-82 |
| CD80- FITC (Clone: 16/10A1) | BD Biosciences | 553768 |
| CD4- PE (Clone: RAM4-5) | ThermFisher Scientific | 12-0042-82 |
| IgA-PE (Clone: mA-6E1) | ThermoFisher Scientific | 12-4204-83 |
| CD5- PerCP-Cy5.5 (Clone:53-7.3) | ThermoFisher Scientific | 45-0051-82 |
| CD25- PerCP-Cy5.5 (Clone: PC61.5) | ThermoFisher Scientific | 45-0251-82 |
| CD45R(B220)-PerCP-Cy5.5 (Clone: RA3-6B2) | ThermoFisher Scientific | 45-0452-1631 |
| CD23- APC (Clone: 2G8) | Southern Biotech | 1585-11 |
| CD8α- Biotin (Clone: 53-6.7) | ThermoFisher Scientific | 13-0081-82 |
| CD138- Biotin (Clone: 281-2) | BD Biosciences | 553713 |
| CD44- PE-Cy7 (Clone: IM7) | BD Biosciences | 560569 |
| CD93- PE-Cy7 (Clone: AA4.1) | ThermoFisher Scientific | 25-5892-82 |
| Streptavidin- PE-Cy7 | BD Biosciences | 557598 |
| IgM- eFluor 450 (Clone: eB121-15F9) | ThermoFisher Scientific | 1020-09 |
| Streptavidin- eFluor™ 450 | ThermoFisher Scientific | 48-4317-82 |
| **Antibodies for IgA Sandwich ELISA** | **SOURCE** | **Cat #** |
| Purified Rat Anti-Mouse IgA | BD Biosciences | 556960 |
| Goat Anti-Mouse IgA - Alkaline Phosphatase | Southern Biotech | 1040-04 |
